# Effects of Seed Processing with Cold Plasma on Growth and Biochemical Traits of *Stevia rebaudiana* Bertoni Under Different Cultivation Conditions: In Soil Versus Aeroponics

**DOI:** 10.3390/plants14020271

**Published:** 2025-01-18

**Authors:** Augustė Judickaitė, Emilija Jankaitytė, Evaldas Ramanciuškas, Laima Degutytė-Fomins, Zita Naučienė, Gediminas Kudirka, Takamasa Okumura, Kazunori Koga, Masaharu Shiratani, Vida Mildažienė, Rasa Žūkienė

**Affiliations:** 1 Faculty of Natural Sciences, Vytautas Magnus University, Universiteto Str. 10, LT-53361 Akademija, Lithuania; 2 UAB “Baltic Freya”, Tulpiu Str. 19, LT-53250 Garliava, Lithuania; 3 Department of Electronics, Faculty of Information Science and Electrical Engineering, Kyushu University, Fukuoka 819-0395, Japan; 4 Plasma-Bio Research Division, Center for Novel Science Initiatives, National Institutes of Natural Sciences, Tokyo 105-0001, Japan

**Keywords:** aeroponics, antioxidant activity, cold plasma, seeds, secondary metabolites, *Stevia rebaudiana* Bertoni, steviol glycosides

## Abstract

This study compared the effects of seed treatment with low-pressure cold plasma (CP) and atmospheric dielectric barrier discharge (DBD) plasma on morpho-biochemical traits in *Stevia rabaudiana* Bertoni plants cultivated by two methods: in soil and aeroponics. We investigated the impact of the treatments on the germination, plant growth, and content of secondary metabolites, namely steviol glycosides (SGs), rebaudioside A (RebA), and stevioside (Stev), as well as phenolic compounds and flavonoids. Seeds were treated for 2, 5, and 7 min with CP or DBD and 5 min with vacuum six days before sowing. All growth parameters in aeroponics exceeded the parameters of seedlings in the corresponding groups cultivated in soil. Seed treatments stimulated SGs biosynthesis in seedlings grown in soil, except for CP7. Although there were no stimulating effects of seed treatments on SGs in aeroponics, overall SG concentrations were considerably higher compared to plants cultivated in soil: the RebA+Stev concentration was 1.8–2-fold higher in the control, V5-, and CP-treated groups, and 1.3-fold higher in the DBD5 and DBD7 groups. Thus, aeroponic cultivation has the potential to improve the growth and synthesis of SGs in stevia, while a combination of aeroponics with seed treatments only increases the content of antioxidants and antioxidant activity.

## 1. Introduction

Sustainable agriculture is directed towards the development of farming systems that are both economically viable and ecologically sustainable [1,2]. Intensive research is focused on enhancing yields and quality of production, using environmentally friendly technologies, and minimizing the intensive use of chemicals for fertilization and plant protection. Among such technologies, the field of plasma agriculture has gained increasing attention and has laid the foundations for a range of applications based on numerous positive effects reported on crop germination and growth (reviewed in [3,4,5,6]).

Non-thermal plasma (NTP) or cold plasma is a non-equilibrium gas discharge plasma, consisting of charged particles, such as ions, free electrons, and neutral particles, including gas molecules, free radicals, and ultraviolet photons [3,7]. Numerous studies have reported that seed irradiation using various NTP sources can decontaminate seeds and stimulate their germination, improve seedling growth, and even increase biomass production or grain yields [3,6]. Strong positive effects of seed irradiation with NTP on plant productivity have been reported for cereal crops [8,9,10,11], legumes [12,13,14], woody [15,16,17], and medicinal plants [13,18,19]. Interestingly, the latter studies reported that seedlings growing from NTP-treated seeds contain higher amounts of biologically active compounds. A strong breakthrough has recently been made in understanding the complexity of the molecular mechanisms underlying plant response to seed processing with NTP [3,6]. It was demonstrated that the physicochemical interaction of NTP with seeds triggers wide-scale changes at the epigenomic, transcriptomic, phytohormonal, and proteomic levels, further leading to changes in numerous biochemical and physiological processes including metabolic and defense enzyme activity, secondary metabolism, photosynthesis, and adaptability to stress on a longer time scale.

Another novel technology extensively explored for sustainable agriculture is indoor farming [20,21] based on soilless plant cultivation. Such farming practices as hydroponics, aeroponics, and aquaponics are valued for their efficient land and water use, yield stability, and environmental control capabilities, since various alternative mediums and systems are used instead of soil for the supply of water, nutrients, and support [22,23]. In contrast to traditional farming methods, vertical farming systems can enhance crop production by utilizing not just the horizontal surface area but also the vertical space above it. As a result, vertical farming has the potential to supply significant quantities of fresh food in densely populated urban areas [21] or in space farming [24]. Indoor farming has expanded quickly within the horticultural sector, and hydroponic technologies have been the most widely used and studied approach for more than a hundred years [25]. Aeroponics is a cutting-edge soilless technique where plants grow on a mist or spray of nutrient-rich media [26]. The advantages of such a cultivation approach are numerous, and include efficient space and water use, efficient nutrient delivery promoting faster and uniform crop growth, optimized root aeration and good access to carbon dioxide, boosting photosynthesis and nutrient absorption, easy monitoring of nutrient levels and pH, allowing precise measurement of nutrient uptake under various conditions, isolation from pathogen pressures, cultivation at latitudes incompatible with certain crops, and utilization of space including disused buildings or tunnels, as well as price stabilization [27,28]. However, not all crops may perform well in aeroponic systems. Therefore, research is needed to adjust cultivation conditions for different species. Nevertheless, aeroponics has been applied successfully to more than 50 species of various crops, including fruits, vegetables, and medicinal plant species [29].

Interestingly, several studies have reported that aeroponic cultivation can induce changes in the amount of biologically active phytochemicals in different parts of medicinal plants. An increased net accumulation of pharmaceutically relevant biologically active secondary metabolites was observed in the roots of aeroponically cultivated *Cannabis sativa* [30]. Six newly identified withanolides (including four with high anticancer activity) were found in the leaves of aeroponically grown *Physalis philadelphica* [31]. The composition of secondary metabolites in the essential oils of aeroponically cultivated *Artemisia pedemontana* was different and exerted a stronger antiparasitic effect compared to the essential oil extracted from greenhouse plants [32]. In light of such findings, the potential of aeroponic cultivation to improve the production of phytochemicals in medicinal plants deserves extended research.

**Figure 1:**
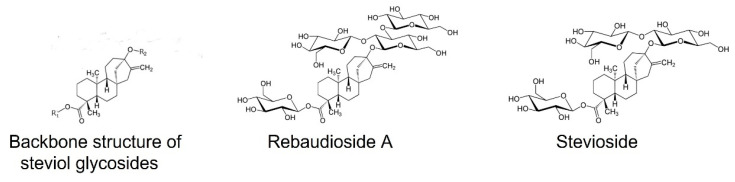
Backbone structure of steviol glycoside and chemical formulas of rebaudioside A and stevioside.

Stevia (*Stevia rebaudiana* Bert. (Bertoni) is a perennial shrub indigenous to Paraguay, South America, and it is nowadays cultivated abundantly in many countries as an economically important source of natural low-calorie sweeteners, namely steviol glycosides (SGs), which are up to 400 sweeter than conventional sugar (sucrose) [33,34]. The stevia market size was estimated at USD 0.84 billion in 2024 and is expected to reach USD 1.36 billion by 2029, growing at a compounded annual rate of 10.12% during the forecast period (2024–2029). However, the number of stevia producers in the market is limited [35]. At least 64 steviol glycosides have been identified in stevia to date [36]. Rebaudioside A (RebA) and stevioside (Stev) are the most abundant steviol glycosides (SGs) responsible for the sweetness of stevia (Figure 1) and account for more than 90% of the total SGs found in dried stevia leaves. RebA is preferred over Stev in stevia products for better taste quality therefore higher RebA/Stev ratio is preferable in stevia products. The licorice off-taste and lingering sweet aftertaste of Stev are eliminated if RebA is present in equal or higher quantities [37].

Numerous studies and clinical trials have demonstrated that, beyond their sweet flavor, stevia extract and steviol glycosides (SGs) provide a range of health benefits, including anti-hypertensive, anti-hyperglycemic, antioxidant, anti-inflammatory, antifungal, antimicrobial, and anti-cariogenic activities (as reviewed in [38]). SGs increase glucose uptake in the myocardium and brain of diabetes patients while reducing glucose accumulation in the liver and kidney. The performance strength of the SGs generally correlated with the number of glucosyl groups in their molecules [36]. Due to these diverse benefits and the absence of side effects with long-term use, stevia-based sweeteners are becoming increasingly popular. In addition to diterpenoid SGs, the non-sweetener components of stevia are rich in phenolic compounds, which further enhance the health benefits of stevia products and increase their overall value.

Based on reported experimental evidence of the multifaceted positive effects of seed treatment with NTP on the performance of various plants [3,4,5,6], we have previously performed studies to explore its potential for improving the germination, growth, and phytochemical composition of stevia [39,40] in both low- and atmospheric-pressure plasma generation systems. The obtained results revealed that short-term pre-sowing treatment of stevia seeds with NTP can effectively enhance the biosynthesis of RebA and Stev. However, it may negatively affect the content of other secondary metabolites, such as phenolic compounds and flavonoids.

In this study, we explored the challenge of aeroponically cultivating stevia. Numerous benefits of the cultivation of stevia in hydroponics, including positive effects on biomass and secondary metabolite accumulation, have been reported. However, aeroponic cultivation has not yet received attention [41]. This plant is known for its poor germination [42]; therefore, we investigated the combined effect of seed treatment with NTP and aeroponic cultivation, evaluating their impact on germination, seedling growth, and the accumulation of valuable secondary metabolites. To our knowledge, no data are available on the combination of aeroponic stevia cultivation with NTP treatments. Taking into account that aeroponic cultivation [30,31,32] and seed processing with NTP [13,18,19] can improve growth and increase the amounts of biologically active compounds in medicinal plants, we aimed to test two working hypotheses: (1) aeroponic cultivation will positively impact growth and valuable secondary metabolites in stevia, and (2) the combination of aeroponics and seed treatment with NTP will enhance the positive effects of aeroponic cultivation on plant productivity.

To test these assumptions, we compared the effects of pre-sowing seed treatment with low-pressure capacitively coupled plasma (CP) and atmospheric-pressure dielectric barrier discharge (DBD) on morphological and biochemical traits in stevia plants cultivated under two different conditions: in soil and aeroponics. We investigated the impact of these treatments on seed germination, seedling biomass, and the content of key secondary metabolites of stevia, namely SGs (RebA and Stev), as well phenolic compounds, flavonoids, and their effects on antioxidant activity. For the first time, we applied a combination of NTP treatment and aeroponic cultivation and found that short pre-sowing treatment of stevia seeds with CP and DBD enhanced the accumulation of RebA and Stev, but only in plants cultivated in soil, compared to the control. In plants cultivated in aeroponics, NTP treatment stimulated the biosynthesis of antioxidant compounds and increased antioxidant activity. Aeroponic cultivation resulted in increased stevia biomass growth compared to soil cultivation.

## 2. Results

### 2.1. Effects on Germination In Vitro and Seedling Emergence in Soil

**Figure 2:**
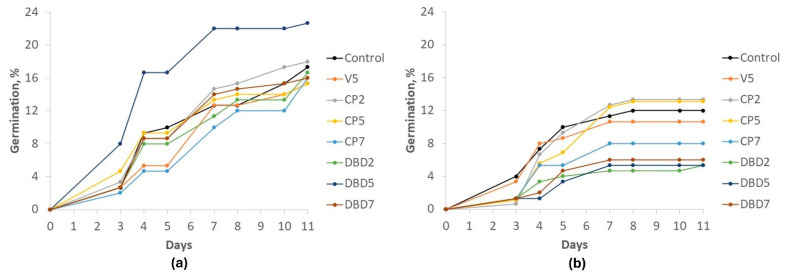
The kinetics of *Stevia rebaudiana* seed germination in vitro (**a**), and seedling emergence in soil (**b**) (Mean, n = 3, SEMs are omitted for clarity).

Based on our previous studies on stevia [39,40] and other plant seeds [13,43,44,45], the seed treatment durations were set at 2, 5, and 7 min for both the CP (this treatment is further abbreviated as CP2, CP5, and CP7, respectively) and DBD (this treatment is further abbreviated as DBD2, DBD5, and DBD7, respectively) treatments. Seed treatment with vacuum, as an additional control for CP, was performed for 5 min (this treatment is further abbreviated as V5). The germination kinetics study in vitro and in soil started 6 days after the DBD and CP treatments. The results are presented in Figure 2a and Figure 2b, respectively. The in-soil germination curve shows a tendency towards a faster germination rate, but has a lower final germination percentage compared to germination in vitro.

Richards plots were used to determine the main indices of germination kinetics for quantitation of differences, and the data are presented in Table 1 and Table 2 for in vitro and seedling emergence in-soil conditions, respectively. Treatment with vacuum, CP, and DBD did not induce statistically significant changes in any of the germination conditions compared to the control.

### 2.2. Effects on Morphometric Parameters

**Figure 3:**
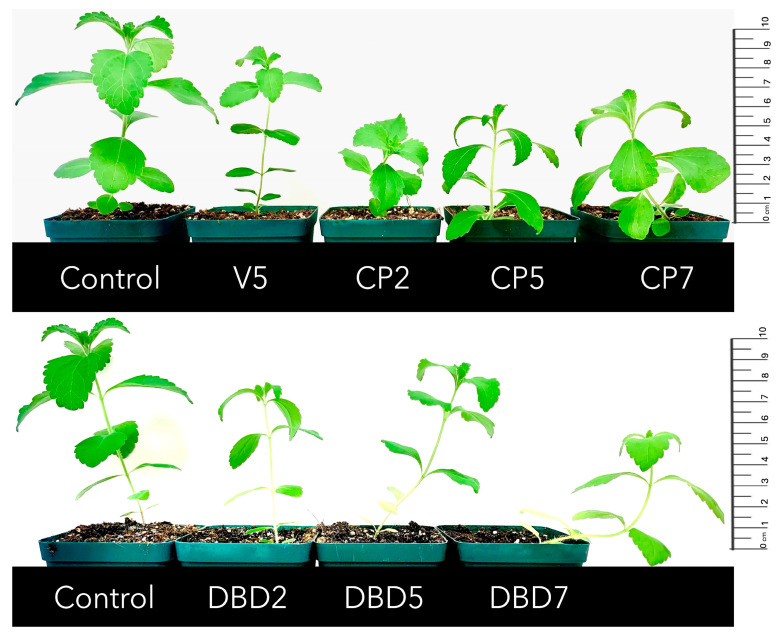
Typical representatives of control and treated groups of stevia plants grown in soil.

**Figure 4:**
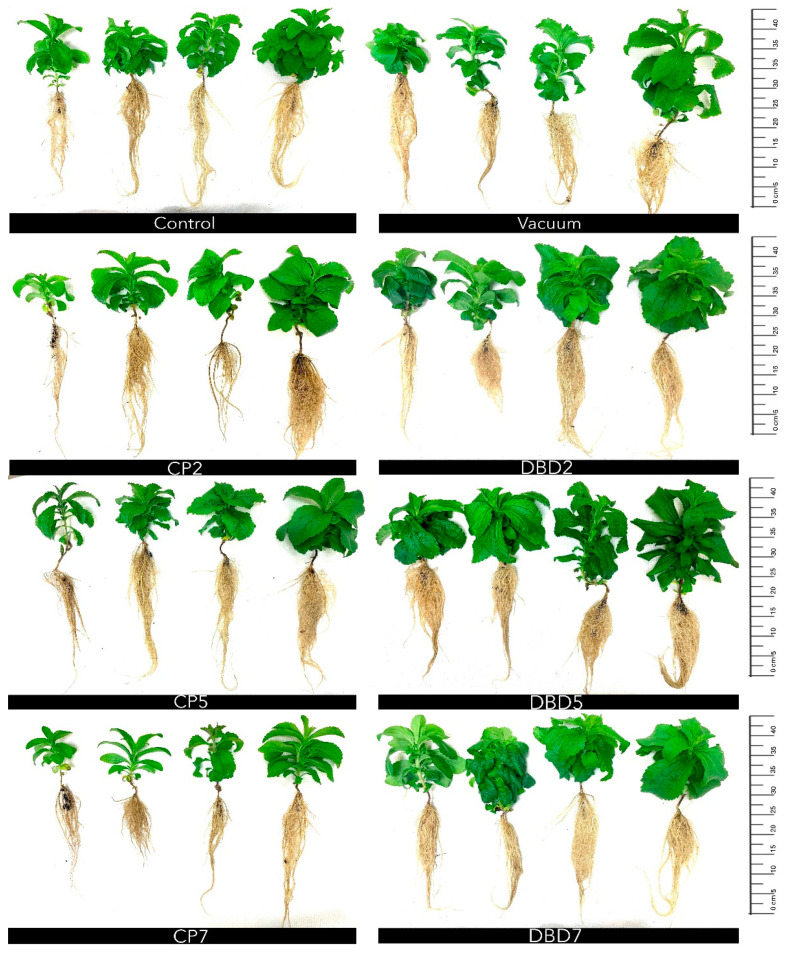
Typical representatives of control and treated groups of stevia plants grown in aeroponics.

Typical stevia plants cultivated in soil and aeroponics are presented in Figure 3 and Figure 4, respectively. The different morphology of plants can be estimated visually. Plants grown in aeroponics were more compact and had denser foliage.

The effects of CP and DBD on morphometric parameters (root length—only for aeroponics, plant height, node number, and leaf number per plant—for both cultivation conditions) of stevia plants are provided in Table 3. CP and DBD did not affect morphometric parameters of stevia cultivated in soil or aeroponics, except for the decreased root length in CP7 in aeroponics. In aeroponics, plants were taller in the control and CP7 groups compared to those grown in soil. In all groups, except for CP7 and DBD2, plants contained more leaves than the corresponding treatment group cultivated in soil.

The effects of CP and DBD on biomass parameters (plant stem fresh and dry weight, leaf fresh and dry weight, leaf dry matter percentage, and leaf/stem ratio of dry weight) of stevia cultivated in soil and aeroponics are presented in Table 4 and Table 5, respectively. In soil, the CP5 and DBD5 groups had a lower leaf dry weight (by 48%), and the CP7 group had a lower leaf dry matter percentage (by 12%). CP and DBD did not affect the morphometric parameters of stevia cultivated in aeroponics, except for a 57% decrease in root fresh weight in the CP7 group and a 67% increase in leaf fresh weight in DBD7. It is worth noting that DBD treatments tended to stimulate biomass growth by 40–67%, determined by leaf fresh weight and 29–65% by leaf dry weight.

The increase in all biomass growth parameters in aeroponics was statistically significant compared to the corresponding group in soil cultivation conditions.

### 2.3. Effects on Concentrations of Steviol Glycosides

**Figure 5:**
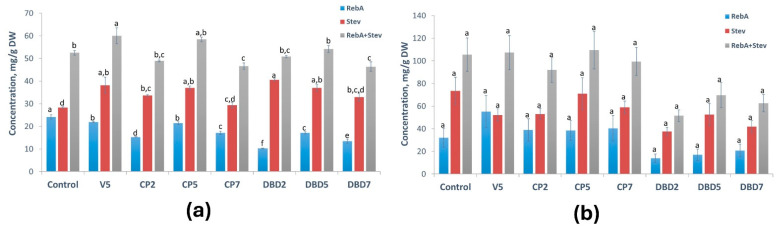
CP- and DBD-induced changes in RebA, Stev, and RebA+Stev concentrations: (**a**) in soil; (**b**) in aeroponics. Mean ± SEM (n = 3), different lowercase letters indicate significant differences (*p* < 0.05, Fisher’s least significant difference (LSD) test).

The changes in the concentration of individual steviol glycosides (RebA, Stev) and their cumulative concentration (RebA+Stev), induced by CP and DBD in the soil- and aeroponics-cultivated stevia, are shown in Figure 5a and Figure 5b, respectively. In soil, stevia grown from treated seeds demonstrated at least one increased parameter, except for CP7 where RebA and RebA+Stev were lower by 29% and 11%, respectively, compared to the control. The strongest stimulation was observed in the V5 and CP5 groups. These treatments increased the Stev concentration by 35% and 31% and the RebA+Stev concentration by 18% and 11%, respectively, compared to the control.

**Figure 6:**
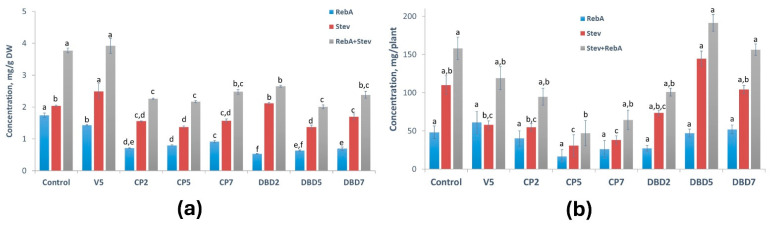
CP- and DBD-induced changes in RebA, Stev, and RebA+Stev amount per plant: (**a**) in soil; (**b**) in aeroponics. Mean ± SEM (n = 6–14), different lowercase letters indicate significant differences (*p* < 0.05, Fisher’s least significant difference (LSD) test).

The stimulating effects of vacuum, CP, and DBD observed in soil-cultivated plants were diminished when the plants were cultivated in aeroponics (Figure 5b). There is a clear downward tendency in all DBD treatment groups. However, it is worth noting that the overall SG concentrations in aeroponically cultivated plants in each experimental group except for DBD2 were higher than in soil-cultivated plants: the RebA+Stev concentration was 1.8–2-fold higher in the control, V5-, and CP-treated groups, and about 1.3-fold higher in the DBD5 and DBD7 groups. In the DBD2 group, the total SG content in aeroponic plants and soil-cultivated plants did not differ. Additionally, when the amount of SG was calculated per plant (Figure 6a,b), this difference increased 10-fold (up to 18–55-fold) compared to plants from the corresponding group grown in soil. Surprisingly, DBD5 surpassed all other treatment groups and the control in aeroponics (Figure 6b).

**Figure 7:**
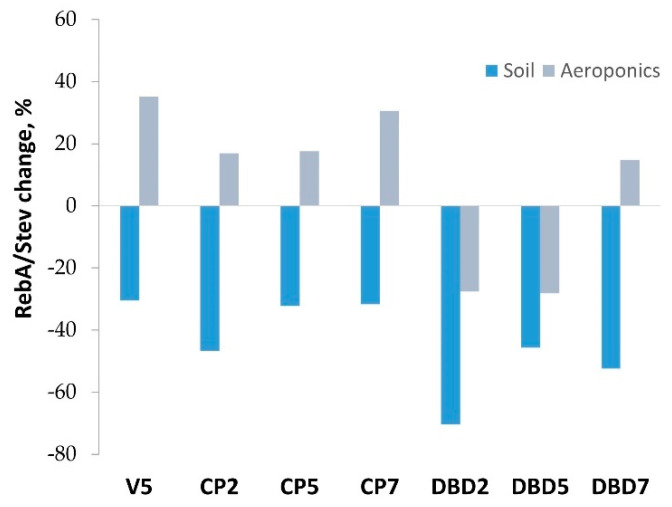
CP- and DBD-induced changes in RebA/Stev ratio compared to the control. Mean (n = 6–14).

Other SG parameters, such as RebA/Stev ratio (a marker of sweetness quality), and the concentration fractions of each SG (RebA/(RebA+Stev) and Stev/(RebA+Stev)), are presented in Table 6. In soil, the RebA/Stev ratio decreased after the treatments, indicating deterioration in the taste quality of the SG mixture. In aeroponics, taste quality was only reduced in the DBD2 and DBD5 groups, where SG concentrations were the lowest (Figure 7).

The 5-min seed treatment with vacuum, which was used as an additional control for low-pressure CP treatment, had the strongest effect on RebA/Stev and increased it up to 0.71 in aeroponics, i.e., by 35% compared to the control group plants (Figure 7). In soil growing conditions, all the seed treatments had a negative effect on RebA/Stev (from 30% in the V5 group up to 70% in the DBD2 group compared to the control).

### 2.4. Effects on Total Phenolic Content, Flavonoid Content, and Antioxidant Activity

**Figure 8:**
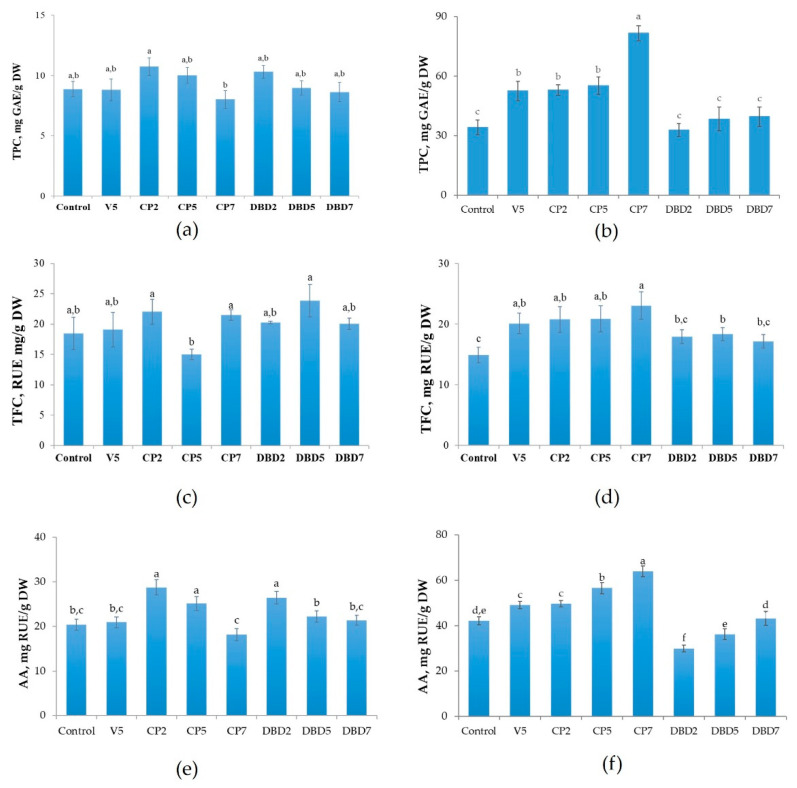
Total phenolic compound (TPC), flavonoids (TFC), and antioxidant activity (AA) in stevia leaves: left panel (**a**,**c**,**e**) plants grown in soil; right panel (**b**,**d**,**f**) plants grown in aeroponics. Mean ± SEM (n = 6–14), different lowercase letters indicate significant differences (*p* < 0.05, Fisher’s least significant difference (LSD) test).

Under soil cultivation conditions, CP and DBD treatment did not affect the total content of phenolic compounds (TPC) and flavonoids (TFC), but CP2, CP5, and DBD2 increased antioxidant activity (AA) by 41%, 23%, and 30%, respectively (Figure 8a,c,e). The effect on AA followed the pattern of changes in TPC with a correlation of r^2^ = 0.97.

In aeroponics, TPC and AA increased in V5 and all the CP groups; AA decreased in the DBD2 group (Figure 8b,d,f). The V5 treatment increased TPC by 50% and the CP treatment increased TPC by 55–205%. In parallel, the V5 treatment increased AA by 16%, the CP treatment increased AA by 18–52%, and the DBD2 treatment decreased AA by 29%. TFC was higher in all the treatment groups by 20–55%, except for DBD2 and DBD7. The effect on AA followed the pattern of changes in TPC, with a correlation of r^2^ = 0.88, and the pattern of changes in TFC, with a correlation of r^2^ = 0.56.

## 3. Discussion

This study aimed to evaluate the potential of aeroponics and pre-sowing seed treatment with different types of NTP, namely CP and DBD, to stimulate the production of the main steviol glycosides and enhance other morphological and biochemical traits in *Stevia rebaudiana*. The results were compared by growing plants under two different conditions: in soil and in a soilless aeroponics system. Available data on the effects of stevia seed treatments with NTP are scarce [39,40] and unsystematic, as studies use different cultivars and plasma configurations. Today, no published data exist on the combined application of seed treatment with NTP and further plant cultivation in aeroponics for any plant species. Only a few studies have investigated the effects of plasma-activated mist [46] or plasma-treated water [47] on plants, such as beans and ginseng, grown in aeroponics.

CP or DBD treatments did not exert an effect on germination in this study. Pre-sowing seed treatment of 5 and 7 min with different device-generated low-pressure CPs increased the germination yield and germination rate in stevia cultivar ‘Criolla’ [39]. The same DBD system used in this study did not stimulate the germination of seeds from different commercial sources, and DBD7 treatment even decreased the final germination percentage [40]. The distinct effects of NTP on seeds of the same species align with previous observations that the effects of seed processing with NTP are highly dependent on cultivar [13,44,45,48], half-sib family [49,50], or even seed coat color [51]. Furthermore, annually changing climatic conditions impact every new seed harvest, and resulting variations in physiological seed status that may significantly influence plant response to NTP treatment. NTP is recognized as a seed dormancy-breaking agent; however, even though stevia seeds are characterized by physiological dormancy, they are often in a non-dormant state [52]. The low germination yields of stevia seeds are not attributed to dormancy but to sterility caused by sporophytic self-incompatibility. The effects of NTP on fertile seeds are determined by the initial ratio between gibberellins and abscisic acid contents [53,54,55]. This ratio, combined with the susceptibility of molecular machinery involved in cell signaling, may modify the effects of plasma on downstream biochemical reactions, leading to variations in the phytohormone ratio.

The morphology of stevia plants cultivated in soil and aeroponics differed visually—plants grown in aeroponics were more compact and had denser foliage. All biomass gain parameters were significantly increased in aeroponics, compared to the corresponding group in soil cultivation conditions. However, seed treatments with CP and DBD did not improve morphometric parameters (height, number of nodes, and leaves) or biomass parameters (stem fresh and dry weight, leaf fresh and dry weight, leaf dry matter percentage, and the leaf/stem ratio of dry weight) except for a higher leaf fresh weight in DBD7 group. On the contrary, the CP5 and DBD5 groups had a lower leaf dry weight, while the CP7 group had a lower leaf dry matter percentage when cultivated in soil, and the CP7 group had a lower root fresh weight when cultivated in aeroponics. Consistent with the results obtained (Table 4), our previous study [40] also reported the tendency towards a DBD-induced biomass decrease in soil-grown plants. In contrast, we observed the tendency of positive DBD treatment effects on biomass gain (increased fresh and dry weight of leaves) (Table 5) when plants were cultivated for 8 weeks in aeroponics. This suggests that repeating the study with longer growing periods would be worthwhile.

Seed treatments stimulated SG biosynthesis in seedlings grown in soil, except for CP7. Although there were no stimulating effects of seed treatments on SGs in aeroponics, overall SG concentrations were considerably higher compared to soil-cultivated plants: the RebA+Stev concentration was 1.8–2-fold higher in the control, V5-, and CP-treated groups, and 1.3-fold higher in the DBD5 and DBD7 groups. Moreover, the SG composition (increased RebA/Stev ratio) in all treatment groups (except for DBD2 and DBD5) indicated a better sweetness quality compared to the control. At the same time, all treatments had a negative impact on the taste quality in plants grown in soil—the RebA/Stev ratio decreased compared to the control. The amounts of SGs calculated per plant were remarkably higher (18–55-fold) in plants from aeroponics, compared to the corresponding group grown in soil, with the DBD5 group plants containing the highest concentrations. These results indicate that aeroponic cultivation has the potential to improve growth and the total SG production in stevia. To determine the best combination of treatment and cultivation conditions, it is important to consider that the key criteria for the economic performance of stevia are the amount of SGs or the qualitative leaf dry weight per unit of area. We did not observe CP- or DBD-induced changes in plant biomass in the 8-week-old plants investigated. However, as the optimal harvest time is at later vegetative stages, the dynamics of these induced changes over a longer time scale should be evaluated. In one of the recent studies, stevia was cultivated approximately for six months in hydroponics before harvesting [56]. It was shown that the SG and biomass yield can be controlled by adjusting the supply of nitrogen and cytokinin (BAP), while maintaining consistent environmental conditions in a hydroponic greenhouse, i.e., a higher average temperature can increase leaf biomass [41].

We found that when stevia is grown in soil, seed treatments with two different types of NTP reveals a trend in secondary metabolite changes. A positive effect on SG synthesis is associated with a neutral [40] or negative [39] effect on TPC and flavonoid levels, as well as a decrease in antioxidant activity. In contrast, CP2, CP5, and DBD2 treatments increased antioxidant activity in this study. This may be partly explained by the different origins and cultivars of stevia seeds. A greater number of positive effects of CP and DBD were observed in aeroponically cultivated plants: CP and vacuum treatment increased all three parameters (TPC, TFC, and AA). DBD5 increased TFC; however, this was not associated with higher AA. Various abiotic physical and chemical stressors typically lead to simultaneous increase in the production of SGs and phenolic compounds [57]. It has been established that seed treatment with NTP induces changes in the secondary metabolism and antioxidant defense in growing seedlings; however, the underlying molecular mechanisms are only beginning to be uncovered (reviewed in [3,6]). Even brief seed irradiation with low-power NTP can activate cellular antioxidant systems, including both an increase in enzyme activities and amounts of ROS-scavenging secondary metabolites [3]. Such effects have been reported for rapeseed [48], brown rice [58], common buckwheat [45,59], maize [14], purple coneflower [18], red clover [13,44], wheat [14], and other plant species. However, the reported effects are highly variable and dependent on numerous factors, such as plant species, plasma generator type, feeding gas, and treatment dose. For example, a TPC increase was observed in spinach seedlings after seed treatment with DBD in nitrogen, while air-based DBD resulted in TPC decrease [60]. A discharge in the air increased the TPC in wheat and rice seedlings, but only after prolonged seed exposure [59]. Moreover, it has been demonstrated that NTP-induced changes in the activities of antioxidant enzymes, TPC, and AA in growing Norway spruce seedlings are dependent both on the genetic line and seedling age [61]. In the case of stevia, future research should aim to identify factors responsible for changes in the biosynthetic pathways of SGs and other secondary metabolites, influenced not only by seed processing with NTP but also by different cultivation conditions.

However, a direct comparison of the two cultivation methods is challenging due to many parameters, such as the absence of symbiotic interactions with root microorganisms, enforced root aeration, and the improved availability of nutrients in aeroponics. Nevertheless, our findings on the potential of aeroponic cultivation to enhance the production of valuable phytochemicals in medicinal plants align with findings of other authors obtained with Himalayan medicinal herb *Picrorhiza kurroa*, cultivated in soil, hydroponic, and aeroponic systems [62]. Plants cultivated in a hydroponic system had better-developed rootlets, while all other morphometric (plant height, leaf length, leaf width, and stem diameter) and physiological (photosynthetic rate, stomatal conductance, transpiration rate) parameters and the content of secondary metabolites picrosides in an aeroponic system were higher compared to plants grown in hydroponic and soil. The authors concluded that the aeroponic system provides the most suitable and sustainable option for producing high-quality *P. kurroa*, required for the preparation of herbal drugs for the industry.

In summary, the obtained results indicate that stevia plants cultivated in aeroponics produced larger biomass and contained higher amounts of secondary metabolites, with SG and TPC levels being 2-fold and 3-fold higher, respectively, compared to plants cultivated in soil. We found that the effects of seed irradiation with NTP on plant growth and biochemical parameters strongly depended on the cultivation method. Consistent with our earlier reports [39], seed treatments had a neutral or even a negative impact on plant growth in soil, but a tendency for a positive DBD effect was observed in aeroponics. Under soil cultivation conditions, seed treatments were effective in changing the amounts of SGs, with particularly strong positive effects from vacuum and CP5 treatments. However, the combination of NTP processing with aeroponics did not result in positive effects on SG levels in the leaves of stevia. In contrast, seed treatments with vacuum and CP increased the amounts of other secondary metabolites (TPC, TFC) and exerted stronger effects on AA in aeroponics, but not in soil.

Thus, our results suggest that aeroponic cultivation of stevia can improve the productivity of this important medicinal plant, particularly due to a substantial increase in SG yield per plant. Further studies are needed to explore the potential of combining aeroponics with seed NTP processing, including the temporal dynamics of the effects or impact of variation in aeroponic cultivation conditions.

## 4. Materials and Methods

### 4.1. Chemicals and Reagents

The standards of rutin, gallic acid, Folin–Ciocalteu phenol reagent, HPLC-grade methanol, 2,2-diphenyl-1-picrylhydrazyl, and ethanol were obtained from Sigma Aldrich (St. Louis, MO, USA). Stevioside and rebaudioside A were purchased from ChromaDex (Los Angeles, CA, USA). HPLC-grade acetonitrile and sodium acetate were obtained from Sharlau Chemie S. A. (Sentmenat, Spain). HCl, sodium carbonate, and acetic acid were purchased from Carl Roth (Karlsruhe, Germany). Hexamethylenetetramine and aluminum chloride were from Thermo Fisher Scientific (Lancashire, UK). The hydroponics media Hydro A and B were obtained from Plagron (Ospel, The Netherlands). All solutions were prepared with ultrapure 18.2 MΩ water from a Watek ultrapure water purification system (Watek Ltd., Ledeč nad Sázavou, Czech Republic).

### 4.2. Plant Material and Experiment Duration

Seeds of the *Stevia rebaudiana* Bertoni cv. Shug A3-6 Hybrid were obtained from the commercial seed source company “EverStevia Canada Inc.” (Toronto, ON, Canada). Seeds were sealed in plastic bags and stored in a refrigerator (+12 °C) under dry conditions in the dark until the experiment date, when they were irradiated with low-pressure (CP) or dielectric barrier discharge (DBD) cold plasma. (21 February 2023). Germination tests were performed from 27 February–10 March 2023, and plants were cultivated from 10 March–5 May 2023.

### 4.3. Seed Treatment with DBD Cold Plasma

**Figure 9:**
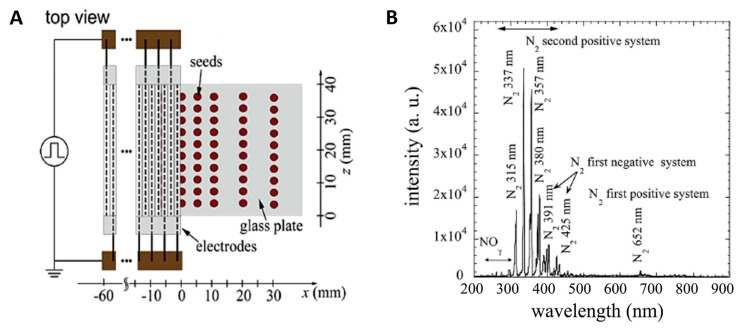
Schematic diagram of the atmospheric-pressure DBD device (**A**) and the optical emission spectrum of the air plasma (**B**).

Plasma irradiation of seeds by a scalable dielectric barrier discharge (DBD) device was carried out as described in [40] and shown in Figure 9A. To create the device, 20 rods of 2 mm in diameter and 60 mm in length were placed in a planar shape with a spacing of 0.2 mm. The rod electrode consisted of a stainless-steel rod of 1 mm in diameter and a ceramic tube of 1 mm in inner diameter and 2 mm in outer diameter. A layer of 300 seeds was placed under the electrode on a glass tray so that the distance between the electrode and the seed surface was 8 mm. Discharges were produced in the air gaps of the electrodes by applying a pulsed voltage of 7 kV (Logy Electric, LHV-09K). The repetition frequency and discharge power density were 14.4 kHz and 3.05 W/cm^2^, respectively, the total power was 4.64 W, and the area of the plasma was 1.52 cm^2^. The optical emission spectrum of the plasma is shown in Figure 9B. Seeds were treated with DBD plasma at room temperature, and the relative humidity of the air was kept at 50 ± 5% by applying an ultrasonic humidifier in a closed chamber where DBD plasma electrodes were placed. Based on some of our previous studies on different plant seeds [13,43,44,45], the chosen duration for seed treatments was 2, 5, and 7 min (this treatment is further abbreviated as DBD2, DBD5, and DBD7, respectively).

### 4.4. Seed Treatment with Low-Pressure Cold Plasma

**Figure 10:**
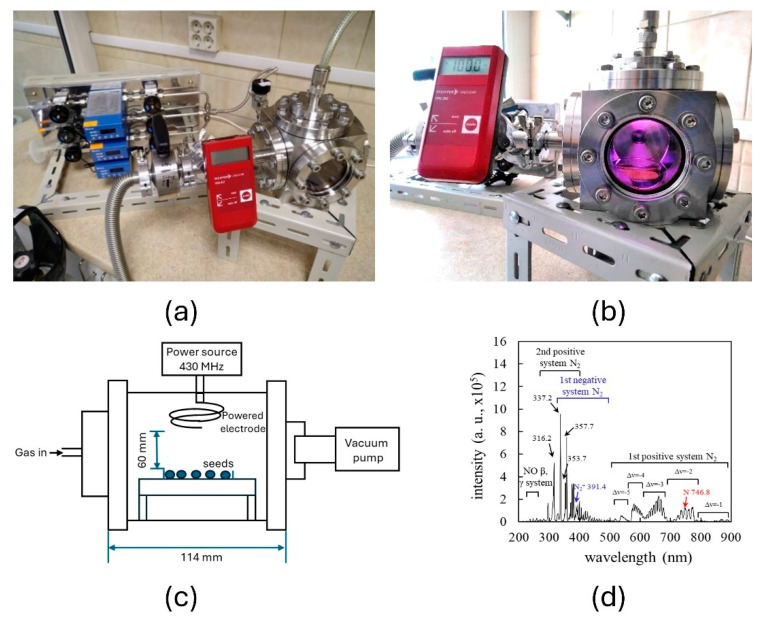
Low-pressure cold plasma device (**a**,**b**) (with ignited plasma in plasma reactor)), scheme of plasma device (**c**), and optical emission spectrum of air plasma (**d**).

Plasma irradiation of seeds by a low-pressure plasma was carried out in a hermetic stainless-steel reactor of capacitively coupled RF discharge (internal diameter—70 mm, external l, h, w—114 mm) (Figure 10). The discharge electrode was a double-turn copper wire coil (wire diameter: 4 mm, coil diameter: 30 mm, height: 20 mm) installed in the upper part of the reactor (Figure 10c). Seeds (300) were arranged in a single layer within a 5 cm diameter glass Petri dish, ensuring no overlap for a uniform treatment effect. The Petri dish was then placed in the reactor 60 mm from the electrode, and the chamber was sealed. A vacuum pump and an airflow controller were used to reach and maintain a constant 100 Pa pressure in the chamber. RF voltage with a frequency of 430 MHz was applied to the powered electrode. The discharge power was 50 W. Optical emission spectra were obtained from RF plasma using a spectrometer (Soma Optics S-2630, Soma Optical Co., Ltd., Tokyo, Japan). For this study, air constituted the gaseous phase, and the airflow was 89 ± 5 mL/min. Seeds subjected to vacuum treatment alone served as an additional control for low-pressure plasma treatment. The duration for seed treatments was 2, 5, and 7 min (this treatment is further abbreviated as CP2, CP5, and CP7, respectively). Seed treatment with vacuum was performed for 5 min (this treatment is further abbreviated as V5).

### 4.5. Seed Germination Test In Vitro and Measurement of Seedling Emergence in Soil

Seed germination was determined in vitro and in soil. In vitro, untreated (control) seeds and seeds exposed to CP and DBD were evenly distributed on 2 layers of filter paper in 90 mm-diameter plastic Petri dishes (3 replicates of 50 seeds each) and watered with 5 mL distilled water. Petri dishes with seeds were placed in a climatic chamber (KK 750, Pol-Eko-Aparatura, Wodzisław Śląski, Poland) with automatically controlled relative humidity (60%), light, and temperature. Alternating light regimes (16 h light, 8 h dark) and a constant temperature of 25 ± 1 °C were maintained in the chamber. Seeds were provided with additional water, if necessary, to prevent drying. A seed was considered germinated when the radicle first emerged. Germinated seeds were counted daily until their number stopped increasing and were then transferred to moistened mineral cotton cubes for aeroponics cultivation.

To estimate the seedling emergence kinetics in soil, seeds were evenly distributed on the humidified substrate surface (3 growth containers (L × D × H 9 × 9 × 10 cm) of 30 seeds each) and watered with 50 mL distilled water. Growth containers were filled with a soil substrate, namely universal peat soil (Durpeta, UAB, Lithuania), consisting of 99.4% upland peat (Sphagnum peat H3–H7), limestone, mineral fertilizers (NPK-14-16-18) with microelements—0.5%, organic substances—92–96%, electrical conductivity (1:1)—0.8–1.4 mS/cm, pH 5.5–6.5 (H_2_O), and mixed with quartz sand (fraction size 0.0–0.4 mm, Anyksciu kvarcas, AB, Lithuania) and perlite (Jukneviciaus kompostas, UAB, Lithuania) at the ratio of 9:1:1.

Growth containers with seeds were placed in a climatic chamber (KK 750, Pol-Eko-Aparatura, Wodzisław Śląski, Poland), and the same conditions were maintained as described above for the germination test in vitro. A seed was considered germinated when the initial emergence of the radicle was observed. Germinated seeds were counted daily until their number stopped increasing.

The effect of CP and DBD on germination was estimated by the induced changes in germination kinetics parameters, derived using the Richards function [63] for the analysis of a germinating seed population [64]. The parameters included Vi (%) (the final germination percentage indicating seed viability), Me (days) (the median germination time (t_50%_) indicating the germination halftime of a seed lot or germination rate), and Qu (days) (the quartile deviation indicating the dispersion of germination time in a seed lot (half of the seeds with an average growth time germinate within the range Me ± Qu)).

### 4.6. Plant Cultivation in Soil

After germination in soil, seedlings were grown for 8 weeks under greenhouse conditions, with a long day photoperiod (16 h light, 8 h dark) produced by artificial high-pressure sodium irradiance, a relative humidity of 60%, and a constant temperature of 25 ± 1 °C. Plants were watered with tap water (pH 7.6–7.9, electrical conductivity 437–508 µS cm^−1^) when needed, maintaining a similar substrate moisture. Plants were grown without additional fertilization.

### 4.7. Plant Cultivation in Aeroponics

**Figure 11:**
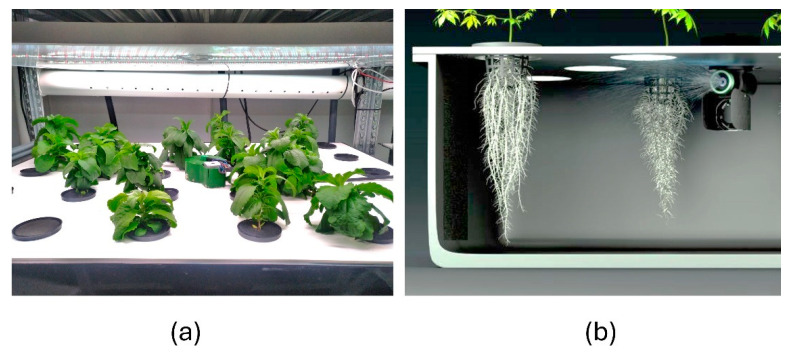
Stevia plants growing in aeroponics (**a**) and illustration of the plant roots and nebulizer (Freya Cultivation Systems) (**b**).

After germination in vitro, stevia seedlings were transferred to 20 × 20 mm Grodan rock wool cubes (Roermond, The Netherlands). Once seedlings developed two true leaves, they were transferred into the aeroponic system within a walk-in, controlled-environment chamber designed to replicate typical vertical farming conditions. Environmental parameters were maintained as follows: a 16 h photoperiod with artificial lighting from a custom LED panel composed of six Samsung Horticulture L2 LED modules, providing a light intensity of 250 µmol m^−2^ s^−1^. Relative humidity was 50 ± 5%, with day and night temperatures of 21 °C and 17 °C, respectively. CO_2_ concentration was maintained at 1000 ppm. An intermittent aeroponic irrigation was carried out using a Nebula R1+ rotational nozzle (Freya Cultivation Systems, Garliava, Lithuania), programmed to spray for 45 s every 495 s. The nutrient solution concentrate, consisting of Plagron’s (Ospel, The Netherlands) Hydro A (NPK 3-0-1 with 4.2% Ca and 0.4% MgO) and Hydro B (NPK 1-3-6 with 1.4% MgO), was diluted at a 1:400 ratio with deionized water. Nutrient solution pH was continuously monitored and adjusted using an acid (HNO_3_) or base (KOH) to maintain a target pH of 5.5–6.5, and the solution’s electrical conductivity was held at 1.56 ± 0.1 mS/cm. A photograph of stevia plants growing in aeroponics and an illustration of the plant roots and nebulizer are shown in Figure 11.

### 4.8. Morphometric Measurements

The plant root length, height, leaf number, stem fresh and dry mass, and leaf fresh and dry mass per plant were measured in 8-week-old plants (n = 6–14).

### 4.9. Plant Material Preparation for Extraction

The leaves were collected from 8-week-old plants and dried at 40 °C for 24 h. Dried leaves were powdered using a batch mill with a disposable grinding chamber (Tube-Mill control, IKA, Staufen, Germany) and stored at room temperature.

### 4.10. Extract Preparation

The leaf powder was mixed with 70% ethanol at a ratio of 1:50 (*w*/*v*). The extraction was carried out in triplicate by sonication for 60 min at 25 °C. The mixture was centrifuged at 16,000× *g* for 10 min, and the supernatant was collected and kept at −20 °C until analysis.

### 4.11. HPLC Analysis of Steviol Glycosides

Steviol glycosides rebaudioside A (RebA) and stevioside (Stev) were separated and quantified using high-performance liquid chromatography (HPLC) [65]. An Agilent 1200 series HPLC system (Agilent Technologies Inc., Santa Clara, CA, USA) with a diode array detector was used. Samples were filtered through a syringe filter with a PVDF membrane (pore diameter 0.22 µm) and separated in a reversed-phase column (Purospher STAR RP-18e 5 µm Hibar 2 × 250 mm, Merck, Germany) with a precolumn. The injection volume was 10 µL at a 70 °C column temperature. Isocratic elution at a flow rate of 0.25 mL min^−1^ with a mobile phase consisting of 70% deionized water acidified with HCl to pH 2.75 and 30% acetonitrile was used for separation with an additional washing step with 50% acetonitrile. RebA and Stev were detected at the wavelength of 210 nm. Calibration was performed by plotting the peak area responses against the concentration values in the concentration range from 1 to 1000 µg mL^−1^ with linear dependence for both analytes. Each analysis was repeated three times, and the mean value was used.

### 4.12. Determination of Total Phenolic Content

Total phenolic content (TPC) was determined using the modified Folin–Ciocalteu method [65]. A total of 0.2 mL of stevia extract was mixed with 1 mL of 0.2 N Folin–Ciocalteu reagent and 0.8 mL 7.5% sodium carbonate solution. After 60 min of incubation in the dark at room temperature, absorbance was measured at 760 nm. Gallic acid was used as a standard, and the results were expressed by mg of gallic acid equivalent (GAE) mg g^−1^ of dry weight (DW).

### 4.13. Determination of Total Flavonoid Content

Total flavonoid content (TFC) was analyzed by using a colorimetric method based on the complexation of phenolic compounds with Al (III) [65]. A total of 80 µL of stevia extract was mixed with 1920 µL of a reagent containing 40% ethanol, 0.7% acetic acid, 0.4% hexamethylenetetramine, and 0.6% aluminum chloride. After 30 min of incubation in the dark at 4 °C, absorbance was measured at 407 nm. Rutin was used as a standard, and the results were expressed by mg of rutin equivalent (RUE) mg g^−1^ of DW.

### 4.14. Determination of Antioxidant Activity

Antioxidant activity (AA) was measured based on the scavenging of the stable 2,2-diphenyl-1-picrylhydrazyl free radical (DPPH), as described [65]. Accordingly, 50 µL of stevia extract was added to 1950 µL of a DPPH solution (25 µg/L, prepared in acetonitrile–methanol–sodium acetate buffer (100 mM, pH 5.5) (1:1:2)). After 15 min of incubation in the dark at room temperature, absorbance was measured at 515 nm. Rutin was used as a standard, and antioxidant activity was expressed by mg of rutin equivalent (RUE) mg g^−1^ of DW.

### 4.15. Statistical Analysis

Statistical analysis of the results was performed using Statistica 7 software (issued by IBM Lietuva, Vilnius, Lithuania to Vytautas Magnus University). One-way analysis of variance (ANOVA) and Fisher’s least significant difference (LSD) test were used to compare the means of treated groups for aeroponics and soil experiments. Results were considered statistically significant at *p* < 0.05. The means of different growing conditions (soil and aeroponics) were compared using Student’s *t*-tests for unpaired samples. Statistical significance of treatment effects was assumed to be statistically significant when *p* < 0.05. All measurements of various parameters between the control and pre-sowing treatment groups were expressed as mean ± SEM.

## Figures and Tables

**Table 1:** Effect of CP and DBD on *Stevia rebaudiana* germination kinetics indices in vitro.

Group	Vi, %	Me, Days	Qu, Days
Control	17.33 ± 2.67 ^a^	4.57 ± 1.13 ^a,b^	1.31 ± 0.70 ^a^
V5	15.33 ± 2.40 ^a^	5.08 ± 0.46 ^a,b^	1.57 ± 0.21 ^a^
CP2	18.00 ± 2.00 ^a^	5.17 ± 0.34 ^a,b^	1.98 ± 0.51 ^a^
CP5	15.33 ± 5.80 ^a^	4.00 ± 0.54 ^b^	1.32 ± 0.65 ^a^
CP7	16.00 ± 4.00 ^a^	6.03 ± 0.90 ^a^	1.78 ± 0.44 ^a^
DBD2	16.67 ± 3.71 ^a^	4.54 ± 0.47 ^a,b^	1.23 ± 0.84 ^a^
DBD5	22.67 ± 3.71 ^a^	3.44 ± 0.42 ^b^	0.79 ± 0.27 ^a^
DBD7	16.00 ± 3.06 ^a^	4.25 ± 0.47 ^a,b^	1.09 ± 0.32 ^a^

Vi, the final germination percentage; Me, the median germination time; Qu, the quartile deviation; mean ± SEM (n = 3), different lowercase letters indicate significant differences (*p* < 0.05, Fisher’s least significant difference (LSD) test).

**Table 2:** Effect of CP and DBD on *Stevia rebaudiana* emergence kinetics indices in soil.

Group	Vi, %	Me, Days	Qu, Days
Control	12.00 ± 3.06 ^a^	3.55 ± 0.34 ^a,b^	0.71 ± 0.24 ^a^
V5	10.67 ± 3.53 ^a^	3.33 ± 0.32 ^b^	0.60 ± 0.23 ^a^
CP2	13.33 ± 6.36 ^a^	4.28 ± 0.22 ^a,b^	0.64 ± 0.24 ^a^
CP5	8.67 ± 7.26 ^a^	4.74 ± 0.10 ^a^	0.64 ± 0.33 ^a^
CP7	8.00 ± 2.0 ^a^	4.15 ± 0.57 ^a,b^	0.83 ± 0.26 ^a^
DBD2	5.33 ± 0.67 ^a^	3.46 ± 0.28 ^a,b^	0.90 ± 0.50 ^a^
DBD5	5.33 ± 0.67 ^a^	4.26 ± 0.69 ^a,b^	0.64 ± 0.46 ^a^
DBD7	6.00 ± 3.74 ^a^	4.21 ± 0.05 ^a,b^	1.11 ± 0.56 ^a^

Vi, the final emergence percentage; Me, the median emergence time; Qu, the quartile deviation; mean ± SEM (n = 3), different lowercase letters indicate significant differences (*p* < 0.05, Fisher’s least significant difference (LSD) test).

**Table 3:** Effect of CP and DBD on morphometric parameters of *Stevia rebaudiana* cultivated in soil and aeroponics.

	Soil			Aeroponics			
	Plant Height, cm	Node Number	Leaf Number	Root Length, cm	Plant Height, cm	Node Number	Leaf Number
Control	12.03 ± 0.62 ^a^	6.55 ± 0.20 ^a,b,c^	13.09 ± 0.62 ^a,b,c^	34.30 ± 5.27 ^a^	8.80 ± 0.69 ^a^*	9.20 ± 0.58 ^a,b^*	17.20 ± 0.80 ^a,b^*
V5	11.93 ± 0.60 ^a^	7.25 ± 0.4 ^a^	14.50 ± 0.82 ^a^	26.65 ± 0.62 ^a,b,c^	10.35 ± 1.75 ^a^	10.17 ± 1.25 ^a^*	20.33 ± 2.50 ^a^*
CP2	9.93 ± 0.76 ^a^	5.93 ± 0.20 ^c^	11.86 ± 0.39 ^c^	25.70 ± 2.49 ^b,c^	10.58 ± 1.74 ^a^	7.80 ± 1.39 ^a,b^*	15.60 ± 2.79 ^a,b^*
CP5	11.47 ± 0.59 ^a^	6.86 ± 0.34 ^a,b,c^	13.71 ± 0.68 ^a,b,c^	32.40 ± 1.97 ^a,b^	9.30 ± 1.15 ^a^	7.40 ± 0.40 ^b^*	14.80 ± 0.80 ^b^*
CP7	12.19 ± 1.28 ^a^	6.90 ± 0.38 ^a,b^	13.80 ± 0.76 ^a,b^	23.70 ± 2.76 ^c^	7.74 ± 0.79 ^a^*	8.00 ± 0.84 ^a,b^	16.00 ± 1.68 ^a,b^
DBD2	10.93 ± 1.55 ^a^	6.75 ± 0.48 ^a,b,c^	13.50 ± 0.96 ^a,b,c^	29.58 ± 3.70 ^a,b,c^	10.17 ± 0.97 ^a^	7.50 ± 0.50 ^b^	15.00 ± 1.01 ^b^
DBD5	10.33 ± 1.03 ^a^	6.00 ± 0.65 ^b,c^	12.00 ± 1.29 ^b,c^	30.00 ± 1.79 ^a,b,c^	10.75 ± 1.5 ^a^	9.00 ± 0.52 ^a,b^*	18.00 ± 1.03 ^a,b^*
DBD7	12.29 ± 1.07 ^a^	6.57 ± 0.53 ^a,b,c^	13.14 ± 1.06 ^a,b,c^	32.33 ± 3.30 ^a,b^	10.80 ± 1.24 ^a^	9.00 ± 0.73 ^a,b^*	18.00 ± 1.46 ^a,b^*

Mean ± SEM (n = 6–14); different lowercase letters indicate significant differences (*p* < 0.05, Fisher’s least significant difference (LSD) test), * statistically significant effect of cultivation conditions (*p* < 0.05); root length was not applicable for the comparison of the cultivation conditions effect.

**Table 4:** Effect of CP and DBD on biomass parameters of *Stevia rebaudiana* plants cultivated in soil.

	Stem FW, g	Stem DW, g	Leaf FW, g	Leaf DW, g	Leaf DM, %	Leaf/Stem (DW)
Control	0.20 ± 0.03 ^a^	0.022 ± 0.004 ^a^	0.74 ± 0.15 ^a^	0.072 ± 0.015 ^a^	9.86 ± 0.26 ^a,b^	3.24 ± 0.16 ^a^
V5	0.19 ± 0.03 ^a^	0.020 ± 0.003 ^a,b^	0.65 ± 0.12 ^a,b^	0.065 ± 0.012 ^a,b^	10.00 ± 0.27 ^a,b^	3.28 ± 0.18 ^a^
CP2	0.13 ± 0.02 ^a^	0.015 ± 0.002 ^a,b^	0.46 ± 0.07 ^a,b^	0.046 ± 0.007 ^a,b^	10.36 ± 0.43 ^a^	3.86 ± 0.83 ^a^
CP5	0.13 ± 0.02 ^a^	0.014 ± 0.002 ^a,b^	0.39 ± 0.08 ^b^	0.037± 0.007 ^b^	9.75 ± 0.55 ^a,b,c^	2.63 ± 0.32 ^a^
CP7	0.17 ± 0.03 ^a^	0.017 ± 0.003 ^a,b^	0.61 ± 0.13 ^a,b^	0.053 ± 0.012 ^a,b^	8.67 ± 0.33 ^c^	3.02 ± 0.34 ^a^
DBD2	0.15 ± 0.04 ^a^	0.014 ± 0.005 ^a,b^	0.56 ± 0.10 ^a,b^	0.052 ± 0.010 ^a,b^	9.32 ± 0.31 ^a,b,c^	4.11 ± 0.41 ^a^
DBD5	0.13 ± 0.03 ^a^	0.011 ± 0.003 ^b^	0.41 ± 0.10 ^a,b^	0.037 ± 0.009 ^b^	8.97 ± 0.17 ^b,c^	4.51 ± 1.38 ^a^
DBD7	0.19 ± 0.04 ^a^	0.020 ± 0.004 ^a,b^	0.57 ± 0.11 ^a,b^	0.051 ± 0.008 ^a,b^	9.44 ± 0.65 ^a,b,c^	2.74 ± 0.26 ^a^

FW—fresh weight, DW—dry weight, DM—dry matter; mean ± SEM (n = 6–14); different lowercase letters indicate significant differences (*p* < 0.05, Fisher’s least significant difference (LSD) test).

**Table 5:** Effect of CP and DBD on biomass parameters of *Stevia rebaudiana* plants cultivated in aeroponics.

	Root FW, g	Root DW, g	Stem FW, g	Stem DW, g	Leaf FW, g	Leaf DW, g	Leaf DM, %	Leaf/Stem (DW)
Control	7.91 ± 2.05 ^a,b,c^	0.79 ± 0.19 ^a,b,c^	1.90 ± 0.81 ^a,b,c^	0.27 ± 0.81 ^a,b^	10.13 ± 3.29 ^b,c^	1.50 ± 0.42 ^a,b,c^	16.00 ± 1.19 ^a,b^	5.89 ± 0.32 ^a^
V5	5.44 ±0.70 ^c,d^	0.50 ± 0.13 ^b,c^	1.31 ± 0.42 ^b,c^	0.19 ± 0.07 ^b^	6.10 ± 1.19 ^c^	1.07 ± 0.33 ^c^	16.13 ± 1.53 ^a,b^	7.02 ± 1.78 ^a^
CP2	6.24 ± 2.19 ^b,c,d^	0.73 ± 0.25 ^a,b,c^	1.19 ± 0.24 ^b,c^	0.24 ± 0.07 ^a,b^	5.77 ± 1.10 ^c^	1.02 ± 0.21 ^c^	17.43 ± 0.47 ^a,b^	4.80 ± 1.02 ^a^
CP5	7.14 ± 1.39 ^b,c,d^	0.70 ± 0.10 ^a,b,c^	1.19 ± 0.20 ^b,c^	0.17 ± 0.03 ^b^	7.07 ± 1.91 ^c^	1.32 ± 0.32 ^b,c^	19.34 ± 1.20 ^a^	7.65 ± 1.20 ^a^
CP7	3.42 ± 0.85 ^d^	0.42 ± 0.07 ^c^	0.64 ± 0.21 ^c^	0.24 ± 0.12 ^a,b^	3.69 ± 1.06 ^c^	0.65 ± 0.23 ^c^	17.01 ± 0.81 ^a,b^	4.21 ± 1.18 ^a^
DBD2	9.10 ± 1.48 ^a,b,c^	0.88 ± 0.14 ^a,b^	2.43 ± 0.31 ^a,b^	0.37 ± 0.05 ^a,b^	14.14 ± 2.48 ^a,b^	1.94 ± 0.31 ^a,b^	14.16 ± 0.84 ^b^	5.42 ± 0.69 ^a^
DBD5	11.56 ± 1.22 ^a^	1.09 ± 0.12 ^a^	2.54 ± 0.49 ^a,b^	0.37 ± 0.08 ^a,b^	16.01± 2.16 ^a,b^	2.26 ± 0.45 ^a,b^	13.77 ± 0.84 ^b^	6.48 ± 0.69 ^a^
DBD7	10.02 ± 1.21 ^a,b^	0.88 ± 0.13 ^a,b^	3.47 ± 1.20 ^a^	0.43 ± 0.13 ^a^	16.90 ± 2.79 ^a^	2.47 ± 0.35 ^a^	15.70 ± 2.14 ^a,b^	7.38 ± 1.83 ^a^

FW—fresh weight, DW—dry weight, DM—dry matter; mean ± SEM (n = 6); different lowercase letters indicate significant differences (*p* < 0.05, Fisher’s least significant difference (LSD) test); the increase in all parameters was statistically significant compared to the corresponding group in soil cultivation conditions; root FW and DW were not applicable for the comparison of the cultivation conditions effect.

**Table 6:** Effect of CP and DBD on *Stevia rebaudiana* leaf steviol glycoside content (mg·g^−1^ of DW) and ratio (mean ± SEM, n = 4) when plants were cultivated in soil.

		Soil			Aeroponics	
	RebA/Stev	RebA/(RebA+Stev)	Stev/(RebA+Stev)	RebA/Stev	RebA/(RebA+Stev)	Stev/(RebA+Stev)
Control	0.85 ± 0.024 ^a^	0.46 ± 007 ^a^	0.54 ± 0.007 ^d^	0.53 ± 0.15 ^a^	0.31 ± 0.08 ^a,b^	0.69 ± 0.08 ^a,b^
V5	0.57 ± 0.073 ^b^	0.37 ± 0.026 ^b^	0.63 ± 0.026 ^c^	0.71 ± 0.20 ^a^	0.37 ± 0.07 ^a,b^	0.63 ± 0.07 ^a,b^
CP2	0.46 ± 0.001 ^c^	0.31 ± 0.001 ^c^	0.67 ± 0.001 ^b^	0.62 ± 0.14 ^a^	0.37 ± 0.05 ^a,b^	0.64 ± 0.05 ^a,b^
CP5	0.58 ± 0.003 ^b^	0.37 ± 0.001 ^b^	0.63 ± 0.001 ^c^	0.62 ± 0.20 ^a^	0.34 ± 0.08 ^a,b^	0.66 ± 0.08 ^a,b^
CP7	0.58 ± 0.003 ^b^	0.37 ± 0.001 ^b^	0.63 ± 0.001 ^c^	0.69 ± 0.34 ^a^	0.46 ± 0.07 ^a^	0.54 ± 0.07 ^b^
DBD2	0.25 ± 0.006 ^d^	0.20 ± 0.004 ^d^	0.80 ± 0.004 ^a^	0.38 ± 0.09 ^a^	0.26 ± 0.05 ^a,b^	0.74 ± 0.05 ^a,b^
DBD5	0.46 ± 0.009 ^c^	0.32 ± 0.004 ^c^	0.68 ± 0.004 ^b^	0.38 ± 0.16 ^a^	0.23 ± 0.08 ^b^	0.77 ± 0.08 ^a^
DBD7	0.41 ± 0.006 ^c^	0.29 ± 0.003 ^c^	0.71 ± 0.003 ^b^	0.60 ± 0.15 ^a^	0.35 ± 0.06 ^a,b^	0.65 ± 0.06 ^a,b^

Different lowercase letters indicate significant differences (*p* < 0.05, Fisher’s least significant difference (LSD) test).

## Data Availability

Data are contained within the article.

## References

[1] Velten S., Leventon J., Jager N., Newig J. (2015). What Is Sustainable Agriculture? A Systematic Review. Sustainability.

[2] Wheaton E., Kulshreshtha S. (2017). Environmental Sustainability of Agriculture Stressed by Changing Extremes of Drought and Excess Moisture: A Conceptual Review. Sustainability.

[3] Bilea F., Garcia-Vaquero M., Magureanu M., Mihaila I., Mildažienė V., Mozetič M., Pawlat J., Primc G., Puač N., Robert E. (2024). Non-Thermal Plasma as Environmentally-Friendly Technology for Agriculture: A Review and Roadmap. Crit. Rev. Plant Sci..

[4] Ishikawa K., Koga K., Ohno N. (2024). Plasma-Driven Sciences: Exploring Complex Interactions at Plasma Boundaries. Plasma.

[5] Pánka D., Jeske M., Łukanowski A., Baturo-Ciésniewska A., Prus P., Maitah M., Maitah K., Malec K., Rymarz D., Muhire J.D.D. (2022). Can Cold Plasma Be Used for Boosting Plant Growth and Plant Protection in Sustainable Plant Production?. Agronomy.

[6] Mildaziene V., Ivankov A., Sera B., Baniulis D. (2022). Biochemical and Physiological Plant Processes Affected by Seed Treatment with Non-Thermal Plasma. Plants.

[7] Misra N., Schlutter O., Cullen P., Misra N.N., Schlutter O., Cullen P.J.M. (2016). Plasma in Food and Agriculture. Cold Plasma in Food and Agriculture: Fundamentals and Applications.

[8] Roy N.C., Hasan M.M., Talukder M.R., Hossain M.D., Chowdhury A.N. (2018). Prospective Applications of Low Frequency Glow Discharge Plasmas on Enhanced Germination, Growth and Yield of Wheat. Plasma Chem. Plasma Process..

[9] Saberi M., Modarres-Sanavy S.A.M., Zare R., Ghomi H. (2018). Amelioration of Photosynthesis and Quality of Wheat Under Non-Thermal Radio Frequency Plasma Treatment. Sci. Rep..

[10] Chanioti S., Katsenios N., Efthimiadou A., Stergiou P., Xanthou Z.-M., Giannoglou M., Dimitrakellis P., Gogolides E., Katsaros G. (2021). Pre-Sowing Treatment of Maize Seeds by Cold Atmospheric Plasma and Pulsed Electromagnetic Fields: Effect on Plant and Kernels Characteristics. Aust. J. Crop Sci..

[11] Recek N., Zaplotnik R., Vesel A., Primc G., Gselman P., Mozetič M., Holc M. (2023). Germination and Growth of Plasma-Treated Maize Seeds Planted in Fields and Exposed to Realistic Environmental Conditions. Int. J. Mol. Sci..

[12] Šerá B., Scholtz V., Jirešová J., Khun J., Julák J., Šerý M. (2021). Effects of Non-Thermal Plasma Treatment on Seed Germination and Early Growth of Leguminous Plants—A Review. Plants.

[13] Mildaziene V., Paužaitė G., Naučienė Z., Zukiene R., Malakauskienė A., Norkeviciene E., Slepetiene A., Stukonis V., Olšauskaite V., Padarauskas A. (2020). Effect of Seed Treatment with Cold Plasma and Electromagnetic Field on Red Clover Germination, Growth and Content of Major Isoflavones. J. Phys. D Appl. Phys..

[14] Filatova I., Lyushkevich V., Goncharik S., Zhukovsky A., Krupenko N., Kalatskaja J. (2020). The Effect of Low-Pressure Plasma Treatment of Seeds on the Plant Resistance to Pathogens and Crop Yields. J. Phys. D Appl. Phys..

[15] Mildaziene V., Pauzaite G., Malakauskiene A., Zukiene R., Nauciene Z., Filatova I., Azharonok V., Lyushkevich V. (2016). Response of Perennial Woody Plants to Seed Treatment by Electromagnetic Field and Low-Temperature Plasma. Bioelectromagnetics.

[16] Pauzaite G., Malakauskiene A., Nauciene Z., Zukiene R., Filatova I., Lyushkevich V., Azarko I., Mildaziene V. (2018). Changes in Norway Spruce Germination and Growth Induced by Pre-Sowing Seed Treatment with Cold Plasma and Electromagnetic Field: Short-Term Versus Long-Term Effects. Plasma Process. Polym..

[17] Šerá B., Šerý M., Zahoranová A., Tomeková J. (2021). Germination Improvement of Three Pine Species (*Pinus*) After Diffuse Coplanar Surface Barrier Discharge Plasma Treatment. Plasma Chem. Plasma Process..

[18] Mildaziene V., Pauzaite G., Naucienė Z., Malakauskiene A., Zukiene R., Januskaitiene I., Jakstas V., Ivanauskas L., Filatova I., Lyushkevich V. (2018). Pre-Sowing Seed Treatment with Cold Plasma and Electromagnetic Field Increases Secondary Metabolite Content in Purple Coneflower (*Echinacea purpurea*) Leaves. Plasma Process. Polym..

[19] Ghasempour M., Iranbakhsh A., Ebadi M.O., Ardebili Z. (2020). Seed Priming with Cold Plasma Improved Seedling Performance, Secondary Metabolism, and Expression of Deacetylvindoline O-Acetyltransferase Gene in *Catharanthus roseus*. Contrib. Plasma Phys..

[20] Klerkx L., Rose D. (2020). Dealing with the Game-Changing Technologies of Agriculture 4.0: How Do We Manage Diversity and Responsibility in Food System Transition Pathways?. Glob. Food Secur..

[21] Benke K., Tomkins B. (2017). Future Food-Production Systems: Vertical Farming and Controlled-Environment Agriculture. Sustain. Sci. Pract. Policy.

[22] Lakhiar I.A., Gao J., Syed T.N., Chandio F.A., Buttar N.A. (2018). Modern Plant Cultivation Technologies in Agriculture Under Controlled Environment: A Review on Aeroponics. J. Plant Interact..

[23] Lakhiar I.A., Gao J.M., Syed T.N., Chandio F.A., Tunio M.H., Ahmad F., Solangi K.A. (2020). Overview of the Aeroponic Agriculture—An Emerging Technology for Global Food Security. Int. J. Agric. Biol. Eng..

[24] Teng Z., Yaguang Luo Y., Pearlstein D.J., Wheeler R.M., Johnson C.M., Wang Q., Fonseca J.M. (2023). Microgreens for Home, Commercial, and Space Farming: A Comprehensive Update of the Most Recent Developments. Ann. Rev. Food Sci. Technol..

[25] Velazquez-Gonzalez R.S., Garcia-Garcia A.L., Ventura-Zapata E., Barceinas-Sanchez J.D.O., Sosa-Savedra J.C. (2022). A Review on Hydroponics and the Technologies Associated for Medium- and Small-Scale Operations. Agriculture.

[26] Lakhiar I.A., Gao J., Xu X., Syed T.N., Chandio F.A., Buttar N.A. (2019). Effects of Various Aeroponic Atomizers (Droplet Sizes) on Growth, Polyphenol Content, and Antioxidant Activity of Leaf Lettuce (*Lactuca sativa* L.). Trans. ASABE.

[27] Eldridge B.M., Manzoni L.R., Graham C.A., Rodgers B., Farmer J.R., Dodd A.N. (2020). Getting to the Roots of Aeroponic Indoor Farming. New Phytol..

[28] Mangaiyarkarasi R. (2020). Aeroponics System for Production of Horticultural Crops. Madras Agric. J..

[29] Gurley T.W. (2020). Aeroponics.

[30] Ferrini F., Donati Zeppa S., Fraternale D., Carrabs V., Annibalini G., Verardo G., Gorassini A., Albertini M.C., Ismail T., Fimognari C. (2022). Characterization of the Biological Activity of the Ethanolic Extract from the Roots of *Cannabis sativa* L. Grown in Aeroponics. Antioxidants.

[31] Xu Y.M., Wijeratne E.K., Brooks A.D., Tewary P., Xuan L.J., Wang W.Q., Sayers T.J., Gunatilaka A.L. (2018). Cytotoxic and Other Withanolides from Aeroponically Grown *Physalis philadelphica*. Phytochemistry.

[32] Sainz P., Andrés M.F., Martínez-Díaz R.A., Bailén M., Navarro-Rocha J., Díaz C.E., González-Coloma A. (2019). Chemical Composition and Biological Activities of *Artemisia pedemontana* subsp. assoana Essential Oils and Hydrolate. Biomolecules.

[33] Gantait S., Das A., Banerjee J. (2018). Geographical Distribution, Botanical Description and Self-Incompatibility Mechanism of Genus Stevia. Sugar Tech..

[34] Lemus-Mondaca R., Vega-Galvez A., Zura-Bravo L., Ah-Hen K. (2012). *Stevia rebaudiana* Bertoni, Source of a High-Potency Natural Sweetener: A Comprehensive Review on the Biochemical, Nutritional and Functional Aspects. Food Chem..

[35] Mordor Intelligence Stevia Market Size & Share Analysis—Growth Trends & Forecasts (2024–2029). https://www.mordorintelligence.com/industry-reports/stevia-market.

[36] Myint K.Z., Chen J.-M., Zhou Z.-Y., Xia Y.-M., Lin J., Zhang J. (2020). Structural Dependence of Antidiabetic Effect of Steviol Glycosides and Their Metabolites on Streptozotocin-Induced Diabetic Mice. J. Sci. Food Agric..

[37] Yadav A.K., Singh S., Dhyani D., Ahuja P. (2011). A Review on the Improvement of Stevia [*Stevia rebaudiana* (Bertoni)]. Can. J. Plant Sci..

[38] Testai L., Calderone V., Wölwer-Rieck U. (2019). Stevia rebaudiana Bertoni. Steviol Glycosides: Cultivation, Processing, Analysis and Applications in Food.

[39] Judickaitė A., Lyushkevich V., Filatova I., Mildažienė V., Žūkienė (2022). The Potential of Cold Plasma and Electromagnetic Field as Stimulators of Natural Sweeteners Biosynthesis in *Stevia rebaudiana* Bertoni. Plants.

[40] Judickaitė A., Venckus J., Koga K., Shiratani M., Mildažienė V., Žūkienė R. (2023). Cold Plasma-Induced Changes in *Stevia rebaudiana* Morphometric and Biochemical Parameter Correlations. Plants.

[41] Partap M., Rattan S., Kainika, Ashrita, Sood A., Kumar P., Warghat A.R., Srivastava D.K., Thakur A.K., Kumar P. (2021). Hydroponic and Aeroponic Cultivation of Economically Important Crops for Production of Quality Biomass. Agricultural Biotechnology: Latest Research and Trends.

[42] Goettemoeller J., Ching A., Janick J. (1999). Seed Germination in *Stevia rebaudiana*. Perspectives on New Crops and New Uses.

[43] Ivankov A., Nauciene Z., Zukiene R., Degutyte-Fomins L., Malakauskiene A., Kraujalis P., Venskutonis P.R., Filatova I., Lyushkevich V., Mildaziene V. (2020). Changes in Growth and Production of Non-Psychotropic Cannabinoids Induced by Pre-Sowing Treatment of Hemp Seeds with Cold Plasma, Vacuum and Electromagnetic Field. Appl. Sci..

[44] Mildaziene V., Ivankov A., Pauzaite G., Naucienė Z., Zukiene R., Degutyte-Fomins L., Pukalskas A., Venskutonis P.R., Filatova I., Lyushkevich V. (2020). Seed Treatment with Cold Plasma and Electromagnetic Field Induces Changes in Red Clover Root Growth Dynamics, Flavonoid Exudation, and Activates Nodulation. Plasma Process. Polym..

[45] Ivankov A., Naučienė Z., Degutytė-Fomins L., Žūkienė R., Januškaitienė I., Malakauskienė A., Jakštas V., Ivanauskas L., Romanovskaja D., Šlepetienė A. (2021). Changes in Agricultural Performance of Common Buckwheat Induced by Seed Treatment with Cold Plasma and Electromagnetic Field. Appl. Sci..

[46] Gao H., Wang G., Huang Z., Nie L., Liu D., Lu X., He G., Ostrikov K.K. (2023). Plasma-Activated Mist: Continuous-Flow, Scalable Nitrogen Fixation, and Aeroponics. ACS Sustain. Chem. Eng..

[47] Song J.S., Jung S., Jee S., Yoon J.W., Byeon Y.S., Park S., Kim S.B. (2021). Growth and Bioactive Phytochemicals of Panax Ginseng Sprouts Grown in an Aeroponic System Using Plasma-Treated Water as the Nitrogen Source. Sci. Rep..

[48] Maruyama-Nakashita A., Ishibashi Y., Yamamoto K., Liu Zhang L., Morikawa-Ichinose T., Sun-Ju Kim S.-J., Hayashi N. (2021). Oxygen Plasma Modulates Glucosinolate Levels Without Affecting Lipid Contents and Composition in *Brassica napus* Seeds. Biosci. Biotechnol. Biochem..

[49] Sirgedaitė-Šėžienė V., Lučinskaitė I., Mildažienė V., Ivankov A., Koga K., Shiratani M., Laužikė K., Baliuckas V. (2022). Changes in Content of Bioactive Compounds and Antioxidant Activity Induced in Needles of Different Half-Sib Families of Norway Spruce (*Picea abies* (L.) H. Karst) by Seed Treatment with Cold Plasma. Antioxidants.

[50] Čėsnienė I., Čėsna V., Miškelytė D., Novickij V., Mildažienė V., Sirgedaitė-Šežienė V. (2024). Seed Treatment with Cold Plasma and Electromagnetic Field: Changes in Antioxidant Capacity of Seedlings in Different *Picea abies* (L.) H. Karst Half-Sib Families. Plants.

[51] Ivankov A., Zukiene R., Nauciene Z., Degutyte-Fomins L., Filatova I., Lyushkevich V., Mildaziene V. (2021). The Effects of Red Clover Seed Treatment with Cold Plasma and Electromagnetic Field on Germination and Seedling Growth Are Dependent On Seed Color. Appl. Sci..

[52] Baskin C.C., Baskin J.M., Baskin C.C., Baskin J.M. (2014). Types of Seeds and Kinds of Seed Dormancy. Seeds. Ecology, Biogeography, and Evolution of Dormancy and Germination.

[53] Degutytė-Fomins L., Paužaitė G., Žukienė R., Mildažienė V., Koga K., Shiratani M. (2020). Relationship Between Cold Plasma Treatment-Induced Changes in Radish Seed Germination and Phytohormone Balance. Jpn. J. Appl. Phys..

[54] Zukiene R., Nauciene Z., Januskaitiene I., Pauzaite G., Mildaziene V., Koga K., Shiratani M. (2019). Dielectric Barrier Discharge Plasma Treatment Induced Changes in Sunflower Seed Germination, Phytohormone Balance, and Seedling Growth. Appl. Phys. Ex..

[55] Attri P., Ishikawa K., Okumura T., Koga K., Shiratani M., Mildaziene M. (2021). Impact of Seed Color and Storage Time on the Radish Seed Germination and Sprout Growth in Plasma Agriculture. Sci. Rep..

[56] Ahmadirad S., Tavakoli A., Mokhtassi-Bidgoli A., Mostashari M.M. (2024). Optimizing Biomass and Steviol Glycoside Yield in Hydroponically Grown Stevia (*Stevia rebaudiana* Bertoni) with Ammonium Nitrate and 6-Benzylaminopurine Concentrations. Sugar Tech..

[57] Ahmad M.A., Javed R., Adeel M., Rizwan M., Yang Y. (2020). PEG 6000-Stimulated Drought Stress Improves the Attributes of In Vitro Growth, Steviol Glycosides Production, and Antioxidant Activities in *Stevia rebaudiana* Bertoni. Plants.

[58] Song J.-S., Kim S.B., Ryu S., Oh J., Kim D.-S. (2020). Emerging Plasma Technology that Alleviates Crop Stress During the Early Growth Stages of Plants: A Review. Front. Plant Sci..

[59] Yodpitak S., Mahatheeranont S., Boonyawan D., Sookwong P., Roytrakul S., Norkaew O. (2019). Cold Plasma Treatment to Improve Germination and Enhance the Bioactive Phytochemical Content of Germinated Brown Rice. Food Chem..

[60] Sera B., Spatenka P., Sery M., Vrchotova N., Hruskova I. (2010). Influence of Plasma Treatment on Wheat and Oat Germination and Early Growth. IEEE Transact. Plasma Sci..

[61] Ji S.H., Choi K.H., Pengkit A., Im J.S., Kim J.S., Kim Y.H., Park Y., Hong J.E., Jung S.K., Choi E.-H. (2016). Effects of High Voltage Nanosecond Pulsed Plasma and Micro DBD Plasma on Seed Germination, Growth Development and Physiological Activities in Spinach. Arch. Biochem. Biophys..

[62] Thakur K., Partap M., Kumar D., Warghat A.R. (2019). Enhancement of Picrosides Content in *Picrorhiza kurroa* Royle ex Benth. Mediated Through Nutrient Feeding Approach Under Aeroponic and Hydroponic System. Ind. Crop. Prod..

[63] Richards F.J. (1959). A Flexible Growth Function for Empirical Use. J. Exp. Bot..

[64] Hara Y. (1999). Calculation of Population Parameters Using Richards Function and Application of Indices of Growth and Seed Vigor to Rice Plants. Plant Prod. Sci..

[65] Blinstrubienė A., Burbulis N., Juškevičiūtė N., Vaitkevičienė N., Žūkienė R. (2020). Effect of Growth Regulators on *Stevia rebaudiana* Bertoni Callus Genesis and Influence of Auxin and Proline to Steviol Glycosides, Phenols, Flavonoids Accumulation, and Antioxidant Activity in Vitro. Molecules.

